# Changes in Quality of Life After Treatment for Non-Melanoma Skin Cancer: A Prospective Cohort Study

**DOI:** 10.3390/jcm15207890

**Published:** 2026-10-13

**Authors:** Ferdi Öztürk, Zehra Neslişah Unan Bacak, Egenaz Şenol Okan, Ümmühan Şeker, Kenan Aydoğan, Serkan Yazici, Süleyman Çeçen, Beyhan Cengiz Özyurt, Hayriye Sarıcaoğlu

**Affiliations:** 1 Department of Dermatology, Faculty of Medicine, Bursa Uludag University, Bursa 16059, Türkiye; egenazsenol@uludag.edu.tr (E.Ş.O.); ummuhanseker@uludag.edu.tr (Ü.Ş.); aydogank@uludag.edu.tr (K.A.); serkanyazici@uludag.edu.tr (S.Y.); hayriye@uludag.edu.tr (H.S.); 2 Department of Dermatology, İznik State Hospital, Bursa 16890, Türkiye; neslisahunan@yahoo.com; 3 Department of Plastic Reconstructive and Aesthetic Surgery, Faculty of Medicine, Bursa Uludag University, Bursa 16059, Türkiye; scecen@uludag.edu.tr; 4 Department of Public Health, Faculty of Medicine, Manisa Celal Bayar University, Manisa 45030, Türkiye; beyhanozyurt@hotmail.com

**Keywords:** non-melanoma skin cancer, quality of life, Dermatology Life Quality Index, Skin Cancer Quality of Life Impact Tool

## Abstract

**Background/Objectives**: Non-melanoma skin cancer (NMSC), particularly basal cell carcinoma (BCC) and squamous cell carcinoma (SCC), may adversely affect quality of life (QoL), especially in cosmetically sensitive areas. This study aimed to identify factors associated with QoL in patients with NMSC and to evaluate changes in dermatology- and skin cancer-specific QoL following treatment. **Methods**: This prospective study included 169 patients with NMSC evaluated at baseline and 3 months after treatment using the Dermatology Life Quality Index (DLQI) and Skin Cancer Quality of Life Impact Tool (SCQOLIT). Factors associated with baseline QoL and changes according to treatment modality and histological subtype were analysed. **Results**: The mean age was 69.2 ± 11.85 years; 82.8% had BCC and 17.2% had SCC. Mean DLQI scores decreased from 2.29 ± 2.81 to 0.34 ± 0.75 and SCQOLIT scores from 4.78 ± 5.53 to 1.20 ± 1.76 at 3 months (both *p* < 0.001). Lower educational level was associated with higher baseline DLQI scores, whereas younger age and higher socioeconomic status were associated with higher and lower baseline SCQOLIT scores, respectively; these associations persisted in sensitivity analyses. The associations of younger age, female sex, and SCC histology with higher DLQI scores were less robust. SCC patients had higher baseline DLQI scores than BCC patients (3.66 ± 4.82 vs. 2.01 ± 2.10, *p* = 0.040), but this difference was no longer statistically significant at 3 months. QoL improved following both surgical and medical treatment, with no significant differences in the magnitude of improvement among surgical or medical treatment types. **Conclusions**: QoL improved significantly within 3 months after NMSC treatment. Different factors were associated with baseline DLQI and SCQOLIT scores, suggesting that these instruments may capture complementary aspects of QoL. Their combined use may provide a more comprehensive assessment of patients with NMSC.

## 1. Introduction

Non-melanoma skin cancer (NMSC) is currently the most prevalent type of malignancy. Although the term encompasses a wide range of tumors, it primarily refers to basal cell carcinoma (BCC) and squamous cell carcinoma (SCC), which together constitute the majority of these lesions. While BCC and SCC are associated with low mortality, their frequent occurrence on the head and neck can lead to substantial morbidity due to cosmetic deformity. Both the presence of NMSC and its treatment may result in disfigurement, potentially causing physical and psychosocial impairment. Therefore, quality of life (QoL) represents a key outcome to consider in the management of NMSC [1].

Several dermatology-specific instruments, including the Dermatology Life Quality Index (DLQI), SF-36, and Skindex-16, have been used in NMSC. However, these instruments may not capture subtle changes at diagnosis or after treatment, supporting the use of skin cancer-specific measures [1,2]. The Facial Skin Cancer Index was developed for head and neck NMSC and has demonstrated validity and reliability, but cannot be applied to lesions at other sites [3].

The Skin Cancer Quality of Life Impact Tool (SCQOLIT) is a skin cancer-specific QoL measure originally developed by Burdon-Jones et al. to assess the quality of life of patients with non-metastatic skin cancers. This 10-item questionnaire uses a 4-point Likert response format, and available evidence supports its reproducibility, validity, and internal consistency [4]. Wali et al. further demonstrated that this tool is feasible for use in dermatology skin cancer clinics among patients with NMSC [5]. Karakok et al. validated the Turkish version of the SCQOLIT [6].

Dermatology Life Quality Index (DLQI) is a widely used quality-of-life measure, validated in Turkish by Öztürkcan et al., and applicable to all dermatological disorders. The scale contains 10 questions assessing the extent to which the disease has influenced patients’ social and physical well-being over the previous week. Higher scores indicate greater impairment in quality of life [7,8].

This study aimed to identify factors associated with QoL in patients with NMSC and assess changes from baseline to 3 months after treatment using both the dermatology-specific DLQI and skin cancer-specific SCQOLIT. By evaluating both instruments concurrently in the same prospective cohort, we sought to characterize dermatology-related impairment alongside skin cancer-specific concerns and examine whether the instruments reveal different patterns of association with patient characteristics. This complementary approach may provide a more comprehensive assessment of patient needs and help inform counselling and follow-up in clinical practice.

## 2. Materials and Methods

### 2.1. Study Design

This was a prospective observational cohort study of adults with histologically confirmed NMSC treated at our tertiary care center between January 2024 and November 2025. Initially, 194 patients were enrolled in the study. During the study period, 25 patients received medical treatment following surgical treatment and were therefore excluded from the analytical cohort to ensure mutually exclusive treatment groups. The final analyses included 169 patients.

This study was approved by the institutional Clinical Research Ethics Committee (approval no. 2023-28/17) and written informed consent was obtained from all the participants. Patients with significant concomitant dermatological disease, other internal malignancies, severe systemic comorbidities or lack of informed consent were excluded. All patients filled in validated Turkish versions of DLQI and SCQOLIT at baseline and third month of treatment. Demographic, clinical and histopathological data were collected through structured clinical interview and medical records, including age, sex, education, comorbidities, smoking and alcohol use, personal and family history of skin cancer, duration of the lesion, histological type, tumor location and size, primary/recurrent status and treatment modality. Education was categorized as below or equal to primary school and above secondary school and lesion duration as 0–6 months, 6–12 months, 1–2, 2–3, 3–4, 4–5, or >5 years.

Tumor location was grouped as head and neck, upper extremity, lower extremity, or trunk and tumor size as <1, 1–4, or >4 cm^2^. Surgery was categorized as primary closure, skin graft, flap repair, or amputation. Medical treatments included cryotherapy, radiotherapy, imiquimod 5%, and vismodegib.

### 2.2. Statistical Analysis

Normality was tested using the Shapiro–Wilk test. Continuous variables are presented as mean ± standard deviation or median (minimum–maximum), as appropriate, and categorical variables as numbers and percentages. Factors associated with baseline DLQI and SCQOLIT scores were assessed using multivariable linear regression, and regression coefficients (B), 95% confidence intervals (CIs), and *p*-values were reported. Regression assumptions were checked. Multivariable linear regression was used to identify factors associated with DLQI and SCQOLIT scores at diagnosis, with age, sex, socioeconomic level, educational level, histological type (BCC vs. SCC), presentation (primary vs. recurrent) and type of surgical reconstruction entered simultaneously (*n* = 145). Because both questionnaires yield bounded, right-skewed scores with a floor at zero, the linear model assumptions were examined: residual normality with Q–Q plots and the Shapiro–Wilk test, homoscedasticity with residual-versus-fitted plots and the modified Breusch–Pagan test, independence with the Durbin–Watson statistic, multicollinearity with variance inflation factors, and influential observations with Cook’s distance (cases with Cook’s D > 4/*n* were flagged). Because the residuals were non-normal and heteroscedastic, each model was re-estimated with heteroscedasticity-consistent (HC3) standard errors and after excluding the flagged influential cases as a sensitivity analysis; associations that were not confirmed in these analyses are reported as exploratory. Paired-samples *t*-tests were used to assess change in DLQI and SCQOLIT scores from baseline to 3 months. A repeated-measures ANOVA was used to examine change over time by surgical and medical treatment type. The within-subject factor was time and the between-subject factor was treatment type. Main effects and time × treatment interactions were evaluated and estimated marginal means were compared using Bonferroni correction. Independent-samples *t*-tests were used to compare DLQI and SCQOLIT scores between BCC and SCC groups with Welch’s correction applied when the assumption of equal variances was violated. Statistical assumptions were checked and deemed appropriate. Analyses were performed with IBM SPSS Statistics for Windows, Version 29.0 (IBM Corp., Armonk, NY, USA) and a two-sided *p* < 0.05 was considered statistically significant.

## 3. Results

The study comprised 169 patients with non-melanoma skin cancer (mean age 69.2 ± 11.85 years; 53.8% male). The demographic, clinical, tumor, and treatment features are included in Table 1.

The most common histological type was basal cell carcinoma (BCC) at 82.8%, followed by squamous cell carcinoma (SCC) at 17.2%. Most of the malignancies were primary tumors in the head and neck region. Surgical intervention was the primary therapeutic approach; however, a subset of patients also had medical treatments.

In multivariable linear regression analysis, younger age (B = −0.060, *p* = 0.003), female sex (B = 0.991, *p* = 0.030), lower educational level (B = −1.898, *p* = 0.004), and SCC histology (B = 1.773, *p* = 0.006) were independently associated with higher baseline DLQI scores. Presentation status (primary vs. recurrent disease) was not significantly associated with DLQI (*p* = 0.799). Patients with SCC experienced significantly greater dermatology-related quality-of-life impairment at diagnosis compared with those with BCC, even after adjustment for demographic and clinical variables (Table 2).

In multivariable linear regression analysis, younger age (B = −0.203, *p* < 0.001) was independently associated with higher baseline SCQOLIT scores, whereas higher socioeconomic status (B = −3.219, *p* = 0.007) was independently associated with lower baseline SCQOLIT scores. There were no significant associations for type of surgical treatment, sex, educational level, pathological diagnosis, or presentation status (all *p* > 0.05) (Table 2).

Both scores at diagnosis were right-skewed with a floor effect (20.0% of patients scored 0 on the DLQI and 25.5% on the SCQOLIT; skewness 4.67 and 1.67). Residuals of both models were non-normal and heteroscedastic (Shapiro–Wilk *p* < 0.001; modified Breusch–Pagan *p* < 0.001 for both); there was no multicollinearity (all VIF ≤ 2.2) or autocorrelation (Durbin–Watson 2.11 and 1.91). One patient with a DLQI score of 26 was highly influential in the DLQI model (Cook’s D = 0.93); no single case was influential in the SCQOLIT model (maximum Cook’s D 0.20). In the sensitivity analysis with HC3 standard errors and after excluding influential cases, the SCQOLIT associations with older age (HC3 95% CI −0.301 to −0.115, *p* < 0.001) and higher socioeconomic level (HC3 95% CI −7.149 to −0.238, *p* = 0.036) were unchanged, as was the DLQI association with lower educational level (HC3 95% CI −3.516 to −0.354, *p* = 0.017). The DLQI associations with female sex (HC3 *p* = 0.040; *p* = 0.079 after excluding influential cases), younger age (HC3 *p* = 0.086) and SCC histology (HC3 *p* = 0.146) were not consistently confirmed and are regarded as exploratory.

Mean DLQI scores decreased from 2.29 ± 2.81 at baseline to 0.34 ± 0.81 at 3 months (*p* < 0.001). Mean SCQOLIT scores decreased from 4.78 ± 5.53 to 1.20 ± 1.76 over the same period (*p* < 0.001) (Table 3).

Among patients receiving medical treatment, dermatology-specific and skin cancer-specific quality-of-life scores significantly improved after treatment. Mean DLQI scores decreased from 2.29 ± 2.51 before treatment to 0.54 ± 1.02 at 3 months after treatment (*p* < 0.001). Similarly, mean SCQOLIT scores decreased from 5.63 ± 5.53 to 1.96 ± 2.31 following treatment (*p* < 0.001) (Table 3).

Also, patients who had surgical treatment had significant improvements in quality-of-life measures after treatment. Mean DLQI scores decreased from 2.29 ± 2.87 pre-treatment to 0.30 ± 0.70 at 3 months post-treatment (*p* < 0.001). In addition, mean SCQOLIT scores significantly decreased from 4.63 ± 5.54 to 1.07 ± 1.63 after treatment (*p* < 0.001) (Table 3).

Repeated-measures ANOVA showed a significant improvement in skin cancer-related quality of life from baseline to 3 months after treatment. In the analysis by surgical treatment type, the overall estimated marginal mean SCQOLIT score decreased from 4.293 at baseline to 1.067 at 3 months (mean difference: 3.226, 95% CI: 1.860–4.593; *p* < 0.001). There was a significant main effect of time (F = 21.783, *p* < 0.001, partial η^2^ = 0.134) but no significant time × surgical treatment type interaction (F = 1.226, *p* = 0.303, partial η^2^ = 0.025) or main effect of surgical treatment type (F = 0.908, *p* = 0.439, partial η^2^ = 0.019).

In the analysis by medical treatment type, the overall estimated marginal mean SCQOLIT score decreased from 5.505 at baseline to 1.659 at 3 months (mean difference: 3.846, 95% CI: 1.377–6.315; *p* = 0.004). A significant main effect of time was also observed (F = 10.558, *p* = 0.004, partial η^2^ = 0.345). No significant time × medical treatment type interaction was observed (F = 0.362, *p* = 0.781, partial η^2^ = 0.052), indicating that no statistically significant difference in the magnitude of change was detected among medical treatment types. The main effect of medical treatment type was also not significant (F = 0.273, *p* = 0.844, partial η^2^ = 0.039).

Overall, quality of life improved significantly from baseline to 3 months after treatment, but no statistically significant differences were shown in the magnitude of improvement between types of surgical and medical treatment.

At baseline, DLQI scores were significantly higher in patients with SCC compared to patients with BCC (3.66 ± 4.82 vs. 2.01 ± 2.10, *p* = 0.040) indicating a higher impairment of dermatology-related quality of life in the SCC group at diagnosis. However, at 3 months after treatment, DLQI scores markedly decreased in both groups, and the difference between groups was no longer statistically significant (0.66 ± 1.04 vs. 0.27 ± 0.66, *p* = 0.066) (Table 4).

No significant differences between BCC and SCC patients were found for SCQOLIT scores at baseline (5.03 ± 6.30 vs. 4.72 ± 5.38, *p* = 0.783) or at 3 months (1.14 ± 1.24 vs. 1.21 ± 1.86, *p* = 0.069). The between-group difference in DLQI observed at baseline was no longer statistically significant at 3 months (Table 4).

## 4. Discussion

The present study showed a significant improvement in quality of life 3 months after treatment in patients with NMSC, as measured by both the DLQI and SCQOLIT. Both medically and surgically treated patients improved, with no significant difference in the magnitude of change according to treatment type. In addition, demographic and clinical variables were independently related to baseline quality of life, but these relationships were different for the DLQI and the SCQOLIT. The results highlight the multifactorial nature of quality of life impairment in NMSC and suggest that dermatology-specific and skin cancer-specific instruments capture partially different aspects of the patient experience.

Sensitivity analyses supported the associations of lower educational attainment with higher DLQI scores and of age and socioeconomic level with SCQOLIT scores. However, the DLQI association with female sex was sensitive to the exclusion of influential observations, while the associations with younger age and SCC histology were not supported consistently across sensitivity analyses. These latter findings should therefore be considered exploratory rather than established independent associations.

The baseline quality-of-life impairment in our cohort was relatively mild with mean scores of 2.29 ± 2.81 for the DLQI and 4.78 ± 5.53 for the SCQOLIT. Both scores, however, dropped substantially after treatment suggesting that even a modest baseline level of impairment may be meaningful to patients. Similar results have been reported earlier. Wali et al. reported a mean baseline SCQOLIT score of 5.05 which decreased to 3.69 at 3 months [5]. Yıldırım et al. reported mean SCQOLIT and DLQI scores of 5.56 and 1.74 respectively in patients with head and neck BCC [9]. These findings are consistent with the hypothesis that NMSC may be associated with a relatively low overall quality of life burden yet still produce measurable disease-specific concerns.

The improvement after treatment is in agreement with previous studies. García-Montero et al. [10] showed that there was a significant improvement in Skin Cancer Index (SCI) scores during follow-up in patients with cervicofacial NMSC and that the degree of improvement was influenced by several demographic and clinical factors. Likewise, Çetinarslan et al. also reported a significant improvement in DLQI scores at 3 months after surgery in BCC and SCC patients as well [11]. Similarly, Kinde et al. reported significant improvement in quality of life after excision and reconstruction of periocular NMSC using the SCI and FACE-Q [12]. Stundys et al. reviewed and concluded that postoperative quality of life is generally improved in patients with surgically treated head and neck BCC or NMSC, but the extent of change varies among populations and measurements tools [13].

Repeated-measures analyses performed separately within the surgical and medical treatment groups showed significant effects of time, but no statistically significant time × treatment type interactions. These findings indicate that differences in the magnitude of QoL change were not detected among the respective treatment types; they do not establish equivalent outcomes. Small and heterogeneous treatment subgroups may have limited statistical power, while non-random treatment allocation introduces potential confounding by indication. These exploratory analyses should therefore be interpreted cautiously. Prior work has also indicated that treatment-related and reconstructive issues could impact quality-of-life outcomes in certain patient populations [10,12].

A total of 82.8% of our cohort were BCC and 17.2% were SCC. Patients with SCC had significantly higher baseline DLQI scores compared to patients with BCC (3.66 ± 4.82 vs. 2.01 ± 2.10). SCC histology was associated with higher DLQI scores in the primary multivariable analysis, but this association was not confirmed in sensitivity analyses and should therefore be considered exploratory. Differences in clinical presentation, lesion characteristics, or anticipated treatment consequences may contribute to this finding, although our data cannot establish the underlying mechanism. In this context, Çetinarslan et al. did not find a significant difference in DLQI scores between BCC and SCC before and after surgery [11]. However, both studies demonstrated significant improvement after treatment, and the difference in baseline DLQI in our cohort was no longer statistically significant at 3 months. García-Montero et al. also found that tumor type was a factor associated with changes in total SCI and emotional domain scores [10]. These findings suggest that histological subtype may be associated with quality of life at diagnosis in some populations, though the association is not consistent across studies and may diminish after treatment.

Notably, the difference between BCC and SCC observed with the DLQI was not confirmed with the SCQOLIT. SCQOLIT scores did not differ significantly between tumor types at baseline or after treatment. This difference could be a result of the different dimensions measured by both instruments. The DLQI addresses symptoms, daily activities and psychosocial functioning while the SCQOLIT is more specific to skin cancer-related concerns. Thus, the greater baseline DLQI impairment of SCC may reflect clinical or functional differences less readily captured by SCQOLIT. Alternatively, patients with BCC and SCC may have similar cancer-related psychosocial concerns. These differing associations suggest that DLQI and SCQOLIT may capture partially distinct aspects of the patient experience.

Younger age was associated with higher SCQOLIT scores, and this association persisted in sensitivity analyses, suggesting greater skin cancer-specific impairment among younger patients. However, the association with DLQI scores was not robust and should be considered exploratory. Younger patients may be more concerned about visible lesions, scarring, social interactions, and the long-term consequences of a cancer diagnosis. Similarly, Kinde et al. reported better quality of life in older age in patients with periocular NMSC [12]. These findings imply that, in younger patients, the psychosocial impact of NMSC may be more detrimental.

Female sex was associated with higher baseline DLQI scores in the primary analysis, but this association was sensitive to the exclusion of influential observations and should be interpreted cautiously. No significant association was found with SCQOLIT scores. Previous findings on sex have been inconsistent. While Çetinarslan et al. found no significant differences according to sex in DLQI scores [11], García-Montero et al. reported that sex affected changes in total SCI and social-aesthetic scores [10]. In addition, Kinde et al. found that being female was a predictor of poorer quality of life in periocular NMSC [12]. These differences could be attributed to differences in tumor location, cosmetic concerns, study populations and domains assessed by different instruments.

Differing associations were also observed for education and socio-economic factors. Lower education level was independently associated with higher baseline DLQI scores while higher socioeconomic status was independently associated with lower SCQOLIT scores. Previous studies have reported similar heterogeneous findings. Çetinarslan et al. found that university graduates had worse scores in DLQI [11]. García-Montero et al. reported a difference in the educational level in the global and emotional quality of life improvement after treatment [10]. Such findings suggest that education and socioeconomic status may play a role in disease awareness, treatment expectations and perceptions of cancer burden, although the direction of such associations appears to differ between populations and instruments.

The predominance of head and neck tumors in our cohort is clinically relevant as lesions in visible areas may disproportionately affect self-perception, social interaction and concerns about scarring and appearance. Beyond treatment category, tumor depth, anticipated defect size, reconstructive requirements, and treatment planning may influence cosmetic and functional outcomes and, consequently, patient-reported QoL, particularly in the head-and-neck region. Popescu et al. reported that preoperative high-frequency ultrasound may support assessment of BCC depth and surgical planning [14]. Although its impact on QoL was not directly evaluated, this approach highlights the potential relevance of detailed preoperative assessment when considering the extent of excision and reconstruction. As reported by García-Montero et al. [10], the degree of quality-of-life improvement in patients with cervicofacial NMSC was related to sex, educational level, marital status, history of depression or anxiety, tumor type, treatment modality, and perceived treatment-related discomfort. In a similar way, Kinde et al. showed that demographic and clinical characteristics can influence the postoperative quality of life in periocular NMSC [12]. Yıldırım et al., however, reported no significant correlation between lesion duration and SCQOLIT or DLQI scores in patients with head and neck BCC [9]. These heterogeneous findings further support the multifactorial nature of quality of life in NMSC.

A key strength of the present study is the concurrent use of DLQI and SCQOLIT, allowing assessment of dermatology-specific and skin cancer-specific dimensions in the same cohort. The different predictors found for the two instruments indicate the added value of this approach. The relevance of QoL assessment extends beyond skin cancer to other dermatological and venereological conditions. Ciccarese et al. examined QoL, mood, and sexual function in patients with sexually transmitted infections, illustrating the importance of evaluating patient experiences beyond physical manifestations [15]. In NMSC, combining dermatology-specific and skin cancer-specific questionnaires may similarly help identify concerns that are not fully captured by routine clinical assessment.

Several limitations should be acknowledged. The 3-month follow-up limits interpretation to short-term outcomes. DLQI scores in this cohort were low and clustered near the floor of the scale, which limits the power of the DLQI model and makes its estimates sensitive to extreme values; the DLQI associations with age and histological type were not robust in sensitivity analyses and should be regarded as hypothesis-generating. The SCQOLIT findings were consistent across all analyses. Small and heterogeneous treatment subgroups may have reduced the ability to detect differences among treatment types; therefore, non-significant interactions should not be interpreted as evidence of equivalent QoL outcomes. The clinical importance of the observed changes was not formally assessed against a minimal clinically important difference for either instrument. Modest baseline impairment and follow-up scores approaching the lower bounds of the scales may have limited measurable improvement and the detection of differences among treatment types. Statistical significance therefore does not establish clinically meaningful improvement. The single tertiary-center setting, predominance of head-and-neck lesions and surgical treatment, and limited representation of some marital and socioeconomic categories restrict generalizability. The exclusion criteria, including the exclusion of patients receiving medical treatment after surgery, may have introduced selection bias and underrepresented patients with greater QoL impairment, although the direction and magnitude of this potential bias cannot be determined. The observational design and non-random treatment allocation preclude causal conclusions and introduce potential confounding by indication. Without an untreated or comparator group, the contribution of treatment cannot be distinguished from regression to the mean, natural symptom resolution, psychological adaptation to diagnosis, or postoperative recovery. Although broad anatomical location and reconstruction categories were recorded, exact head-and-neck location, tumor visibility, reconstructive complexity, margin status, postoperative complications, pain, functional impairment, and cosmetic outcomes were not systematically assessed in sufficient detail. These omissions may result in residual confounding in both the regression and treatment-type analyses.

## 5. Conclusions

QoL improved during the 3-month period following treatment for NMSC, as measured by both the DLQI and SCQOLIT. Improvement was observed following both surgical and medical treatment, with no significant differences in the magnitude of improvement among treatment types. Nevertheless, treatment heterogeneity, small subgroup sizes, and confounding by indication limit these analyses. Removing overlapping cases does not eliminate these limitations. Lower educational attainment was associated with greater dermatology-related impairment, while age and socioeconomic status were associated with skin cancer-specific impairment. Associations of DLQI scores with age, sex, and histological subtype were less robust. These findings support a multidimensional approach to quality-of-life assessment in NMSC and suggest that combining dermatology-specific and skin cancer-specific patient-reported outcome measures may provide a more comprehensive understanding of the patient experience.

## Figures and Tables

**Table 1:** Demographic, clinical, tumor, and treatment characteristics of the study population (*n* = 169).

Characteristic	*n*	%
Demographic and clinical characteristics		
Age, years, mean ± SD	69.20 ± 11.85	—
Sex		
Male	91	53.8
Female	78	46.2
Educational level		
Primary or less	91	53.8
Secondary or higher	78	46.2
Socioeconomic status		
Low	72	42.6
Middle	96	56.8
High	1	0.6
Employment		
Inside home	113	66.9
Outside home	56	33.1
Marital status		
Unmarried	3	1.8
Married	166	98.2
Comorbid conditions		
None	26	15.5
One or more	143	84.5
History of depression or anxiety		
Yes	36	21.3
No	133	78.7
Personal history of skin cancer		
Yes	45	26.6
No	124	73.4
Family history of skin cancer		
Yes	27	16.0
No	142	84.0
Tumor characteristics		
Histological diagnosis		
Basal cell carcinoma	140	82.8
Squamous cell carcinoma	29	17.2
BCC subtype (*n* = 140)		
Superficial	7	5.0
Nodular	90	64.3
Infiltrative	24	17.1
Morpheaform	2	1.4
Basosquamous	17	12.1
SCC subtype (*n* = 29)		
Poorly differentiated	-	-
Well differentiated	29	100.0
Tumor location		
Head and neck	155	91.7
Upper extremity	4	2.4
Lower extremity	2	1.2
Trunk	8	4.7
Presentation status		
Primary	156	92.3
Recurrent	13	7.7
Lesion size		
<1 cm^2^	69	40.8
1–4 cm^2^	71	42.0
>4 cm^2^	29	17.2
Lesion duration		
0–6 months	43	25.4
6–12 months	30	17.8
1–2 years	34	20.1
2–3 years	26	15.4
3–4 years	6	3.6
4–5 years	5	3.0
>5 years	25	14.8
Treatment characteristics		
Treatment		
Surgery	145	85.8
Cryotherapy	13	7.7
Radiotherapy	4	2.4
Imiquimod 5%	4	2.4
Vismodegib	3	1.8
Type of surgery (*n* = 145)		
Primary closure	45	31.0
Skin graft	54	37.2
Flap	43	29.7
Amputation	3	2.1

Data are presented as *n* (%) unless otherwise indicated. SD, standard deviation; BCC, basal cell carcinoma; SCC, squamous cell carcinoma.

**Table 2:** Multivariable linear regression analyses of factors associated with baseline DLQI and SCQOLIT scores.

Variable	DLQI B (95% CI)	β	*p* Value	SCQOLIT B (95% CI)	β	*p* Value
Age	−0.060 (−0.09 to −0.021)	−0.236	**0.003**	−0.203 (−0.276 to −0.130)	−0.418	**<0.001**
Female sex	0.991 (0.098 to 1.884)	0.173	**0.030**	0.713 (−0.962 to 2.389)	0.065	0.401
Educational level	−1.898 (−3.168 to −0.629)	−0.330	**0.004**	−1.043 (−3.425 to 1.138)	−0.094	0.388
Socioeconomic status	—	—	—	−3.219 (−5.527 to −0.911)	−0.304	**0.007**
Surgical treatment type	—	—	—	−0.540 (−1.546 to 0.466)	−0.081	0.291
Pathological diagnosis (SCC vs BCC)	1.773 (0.516 to 2.947)	0.221	**0.006**	0.582 (−1.698 to 2.862)	0.038	0.615
Presentation status (recurrent vs. primary)	0.209 (−1.413 to 1.830)	0.020	0.799	−0.784 (−3.829 to 2.258)	−0.094	0.611

B, unstandardized regression coefficient; β, standardized regression coefficient; CI, confidence interval; DLQI, Dermatology Life Quality Index; SCQOLIT, Skin Cancer Quality of Life Impact Tool; BCC, basal cell carcinoma; SCC, squamous cell carcinoma. —, variable not retained in the final multivariable model. Statistically significant *p* values are shown in bold.

**Table 3:** Changes in DLQI and SCQOLIT scores from baseline to 3 months in the overall study population and according to treatment modality.

Study Group	Outcome	Baseline, Mean ± SD	3 Months, Mean ± SD	*p* Value
Overall population (*n* = 169)	DLQI	2.29 ± 2.81	0.34 ± 0.75	<0.001 *
	SCQOLIT	4.78 ± 5.53	1.20 ± 1.76	<0.001 *
Medical treatment (*n* = 24)	DLQI	2.29 ± 2.51	0.54 ± 1.02	<0.001 *
	SCQOLIT	5.63 ± 5.53	1.96 ± 2.31	<0.001 *
Surgical treatment (*n* = 145)	DLQI	2.29 ± 2.87	0.30 ± 0.70	<0.001 *
	SCQOLIT	4.63 ± 5.54	1.07 ± 1.63	<0.001 *

Data are presented as mean ± standard deviation. * Paired-samples *t*-test. DLQI, Dermatology Life Quality Index; SCQOLIT, Skin Cancer Quality of Life Impact Tool. Treatment groups were mutually exclusive and included patients receiving surgical treatment only (*n* = 145) or medical treatment only (*n* = 24). Statistically significant *p* values are shown in bold.

**Table 4:** Comparison of DLQI and SCQOLIT scores between patients with basal cell carcinoma and squamous cell carcinoma at baseline and 3 months.

Outcome	BCC (*n* = 140), Mean ± SD	SCC (*n* = 29), Mean ± SD	*p* Value
DLQI at baseline	2.01 ± 2.10	3.66 ± 4.82	0.040 *
DLQI at 3 months	0.27 ± 0.66	0.66 ± 1.04	0.066 *
SCQOLIT at baseline	4.72 ± 5.38	5.03 ± 6.30	0.783 *
SCQOLIT at 3 months	1.21 ± 1.86	1.14 ± 1.24	0.069 *

Data are presented as mean ± standard deviation. * Independent-samples *t*-test. BCC, basal cell carcinoma; SCC, squamous cell carcinoma; DLQI, Dermatology Life Quality Index; SCQOLIT, Skin Cancer Quality of Life Impact Tool. Statistically significant *p* values are shown in bold.

## Data Availability

The data that support the findings of this study are available from the corresponding author upon reasonable request.

## Abbreviations

NMSC	Non-melanoma skin cancer
BCC	Basal cell carcinoma
SCC	Squamous cell carcinoma
QoL	Quality of life
DLQI	Dermatology Life Quality Index
SCQOLIT	Skin Cancer Quality of Life Impact Tool
